# Progress in Gynæcology

**Published:** 1899-12-02

**Authors:** 


### PROGRESS IN GYNÆCOLOGY.

**The Menopause.**—A great deal of attention has lately been given to this important subject, and the natural compared with the artificial menopause was the subject of a very instructive paper by Bland Sutton[1] at the meeting of the British Medical Association this year. By artificial menopause is meant the menopause produced by removal of the uterine appendages, and that produced by removal of the uterus without removal of the appendages. The ovary, apart from being ovigerous, fulfils other duty; it belongs to the class of ductless glands of which the thyroid is the type, and it must also be included in that peculiar group of temporary glands of which the thymus is the best representative. Thus the ovary is a temporary and ductless gland, its period of activity being coincident with menstrual life; the incidence of which is termed puberty, and its natural cessation we conveniently call the menopause. The uterus is secondary, and certainly subservient to the ovaries. Its period of greatest development is coincident with that of the ovaries. Structurally it is a hollow muscle, an incubation chamber for the reception and development of oösperms. Its chief peculiarity among hollow muscles, apart from serving as a temporary resting-place for the embryo, is the remarkable cyclic changes exhibited during menstrual life by the mucous membrane lining that portion of the organ known as the cavity of the uterus. That these extraordinary changes depend on the ovaries, and not on the uterus, is proved by the fact that after the complete removal of both ovaries the regular periodical decidual formation and loss of blood are permanently arrested. The normal menopause is not only declared by a cessation of the monthly loss of blood from the uterus, but by the peculiar cutaneous phenomenon known as "flushing," and the occurrence of nerve disturbance shows itself also by such signs as irritability of mind, nervousness, and occasionally erotomania. Although removal of the uterus from a mature woman produces amenorrhœa and sterility it does not induce a complete menopause. On the other hand, complete removal of both ovaries from a sexually mature woman is followed by amenorrhœa, sterility, and in a fair proportion of cases by "flushings." These signs do not supervene after removal of the uterus alone, and it was with the view of avoiding the production of an artificial menopause that induced Bland Sutton to remove the uterus in many grave conditions in preference to the sacrifice of both ovaries, and in performing hysterectomy to spare at least one ovary.

**Malignant Disease of the Uterus.**—More Madden,[2] in a paper on the treatment of uterine cancer, insists on the vital importance of its early recognition. In the majority of cases the disease originates in the cervix, and remains localised therein for a sufficient time to admit of effectual curative treatment by the early removal of the affected parts. He thinks that it may very frequently be recognised before the occurrence of any of its generally described objective symptoms, such as pain, hæmorrhage, or fœtid discharge, by careful local examination in all cervical lesions. If such examination be made, and early malignant disease is present, the margin of the os uteri will generally be found hard and often red, and in the situation of the muciparous glands small and distinctly defined projections, somewhat like grains of shot under the mucous membrane, will be discovered, pressure on which causes pain and nausea. The circumference of the os feels indurated, and is of a deep crimson colour, or if eroded presents some slightly projecting points which bleed readily. These nodules, when excised, show microscopically malignant characteristics. Eden[3] points out that there is one condition which gives rises to nodules which might possibly be mistaken for early cancer of the cervix, viz., the occurrence of Nabothian follicles; these are retention cyst in the substance of the cervix developed from the deep parts of the cervical glands. But they are smooth instead of being rough, translucent instead of being opaque, do not break away or bleed on being touched, and if you puncture them glairy mucus escapes. As regards treatment, More Madden thinks that in the modern treatment of uterine cancer too exclusive attention is given to hysterectomy as a curative procedure in contradistinction to early amputation of the cervix, at the same time fully recognising the fact that in some instances when the disease has distinctly originated in the body or fundus of the uterus, and in more advanced cases of disease of the cervix, hysterectomy may be an unavoidable necessity. Dr. Reynier,[4] of Paris, advocates total abdominal hysterectomy in uterine cancer. The operation permitted, he argued, a more thorough inspection and removal of the lesion than by the vaginal route. Dr. Jacobs, of Brussels, agrees that the abdominal route is the only proper one for a radical operation. Dr. Doyen[5] thinks that in many of the cases in which abdominal hysterectomy is recommended, the disease is so advanced that operation is contra-indicated. Jessett[6] maintains that there are very few operative cases which cannot be done per vaginam. Cases from an operative point of view should, he says, be classified according to their situation: (1) The os; (2) the inner part of the cervix; (3) the fundus. Those of the second group are the most unfavourable for operation, on account of early extension. Those of the third class are especially encouraging, involvement of glands being usually late. Penrose[7] calls attention to the favourable results of operation for cancer of the fundus uteri, which are superior, he thinks, to the results in operation for malignant disease in any other part of the body.

**Fibromyomata of the Uterus.**—Lewers,[8] discussing the treatment of these tumours, says that in some cases there are no symptoms, and in these no treatment is required. In other cases the symptoms can be kept in check by prolonged medicinal and dietetic treatment so long as the patient is content to employ it. When, however, the symptoms, more especially the bleeding, persist in spite of treatment, the next step usually is to dilate the cervix, and to ascertain whether any tumour is so situated as to be capable of being removed by the vagina. If there is nothing that can be removed in this way, dilatation of the cervix, followed by curetting and the application of tincture of iodine to the endometrium, often improves matters, for a time at least. Finally, if all the above measures fail, or if in the first instance the symptoms are due to mechanical causes, owing to the great size of the tumour, there remains for consideration some form of major operation. Removal of the appendages is open to many objections, and only suitable for cases where the tumour is of moderate size. More certainly effective is removal of the uterus and tumours. The cervix may either be left and treated by the intraperitoneal method, or the whole uterus, cervix as well as body, may be removed by abdominal panhysterectomy. Landau,[9] writing on the same subject, thinks that the dangers of myomata are usually underrated; besides hæmorrhage, inflammatory and suppurative processes in the appendages or peritoneum are frequent complications. The good effect generally attributed to the menopause is seen only in exceptional cases. Too often the presence of the fibroid postpones the cessation of menstruation indefinitely, so that the menorrhagia continuing the operation has to be performed after all with less chance of success. Considering these and other dangers a radical operation should, he thinks, be performed more often than is the rule, especially in women who have to earn their own living.

**Treatment of the Acute form of Gonorrhœal Salpingitis.**—W. J. Taylor[10] points out that in the acute forms of gonorrhœal salpingitis, when specific vaginitis and endometritis are also present, and in gonorrhœal vaginitis when it may still be possible to limit the upward spread of the disease, local treatment is of very great, and indeed of primary importance. As regards the gonococcus the strongest and best local germicides known are the nitrate of silver, the perchloride of mercury, and ichthyol. In all cases of acute gonorrhœal salpingitis in which the uterus and vagina are also affected he recommends a vaginal suppository of ichthyol (10 per cent.) every night and a douche of crude acetic acid during the day. If, as only very rarely happens, the patient comes almost immediately after exposure to contagion it may be advisable to disinfect the vulva, vagina, and cervix manually as in a vaginal cœliotomy. No unnecessary examinations should be made, and the use of the sound should be forbidden as most dangerous.

[1] Bland Sutton, Brit. Med. Jour., Oct. 14, 1899. [2] More Madden, Brit. Med. Jour., Oct. 14, 1899. [3] T. W. Eden, Clin. Jour., Aug. 2, 1899. [4] Dr. Reynier, Med. Rec., Aug. 26, 1899. [5] Doyen, Med. Rec., Aug. 26, 1899. [6] Jessett, Med. Rec., Aug. 26, 1899. [7] Penrose, Annals of Surgery, June, 1899. [8] Lewers, Lancet, July 8, 1899. [9] Landau, Brit. Med. Jour., Aug. 26, 1899. [10] W. J. Taylor, Clin. Jour., Sept. 1899.

